# Public Health 3.0: A Call to Action for Public Health to Meet the Challenges of the 21st Century

**DOI:** 10.5888/pcd14.170017

**Published:** 2017-09-07

**Authors:** Karen B. DeSalvo, Y. Claire Wang, Andrea Harris, John Auerbach, Denise Koo, Patrick O’Carroll

**Affiliations:** 1 New Orleans, Louisiana; 2 Columbia University, Mailman School of Public Health, New York, New York; 3 Washington, DC; 4 Trust for America’s Health, Washington, DC; 5 Atlanta, Georgia; 6 The Task Force for Global Health, Decatur, Georgia

## Abstract

Public health is what we do together as a society to ensure the conditions in which everyone can be healthy. Although many sectors play key roles, governmental public health is an essential component. Recent stressors on public health are driving many local governments to pioneer a new Public Health 3.0 model in which leaders serve as Chief Health Strategists, partnering across multiple sectors and leveraging data and resources to address social, environmental, and economic conditions that affect health and health equity. In 2016, the US Department of Health and Human Services launched the Public Health 3.0 initiative and hosted listening sessions across the country. Local leaders and community members shared successes and provided insight on actions that would ensure a more supportive policy and resource environment to spread and scale this model. This article summarizes the key findings from those listening sessions and recommendations to achieve Public Health 3.0.

**Editor’s Note: **This article is a joint publication initiative between *Preventing Chronic Disease* and *NAM Perspectives*.

## Introduction

The United States has made enormous progress during the past century in improving the health and longevity of its population through public health interventions and high-quality clinical care. In 2015, life expectancy at birth was 78.8 years, 10 years longer than in the 1950s (1). Smoking prevalence rates among adults and teenagers are less than half what they were 50 years ago (2). The proportion of people without health insurance is at a historic low of 8.8% (3). Health reform efforts have also improved health care quality and slowed the growth rate of health care costs.

However, this success falls short of ensuring that everyone in America can achieve an optimal and equitable level of health. The Centers for Disease Control and Prevention (CDC) recently reported that the historical gain in longevity in the United States has plateaued for 3 years in a row (4). Racial and ethnic disparities persist across many health outcomes and conditions, including life expectancy, infant mortality, and exposure to environmental pollutants (5). The gap in life expectancy between people with the highest and lowest incomes is narrow in some communities but wide in others (6). By mapping life expectancies in several cities across the United States, researchers illustrated that this metric can differ by as much as 20 years in neighborhoods just a few miles apart (7). These data suggest that investing in safe and healthy communities matters, especially for the most disadvantaged populations (8). However, many of these challenges require community-based interventions beyond health care. Indeed, today a person’s zip code may be a stronger determinant of health than is his or her genetic code (7,9).

To solve the fundamental challenges of population health, we must address the full range of factors that influence a person’s overall health and well-being. Education, safe environments, housing, transportation, economic development, access to healthy foods — these are the major social determinants of health, comprising the conditions in which people are born, live, work, and age (10). Fortunately, many pioneering communities across the country are already working to improve health by influencing these determinants in a positive way. From Nashville, Tennessee, to Manchester, New Hampshire, to Harris County, Texas, and the Shoalwater Bay Indian Tribe in Washington, community leaders have built coalitions to improve educational attainment, promote economic opportunity, ensure community safety, and build environments that promote mental health and community engagement.

## Key Influence of the Social Determinants of Health

**Figure 1:**
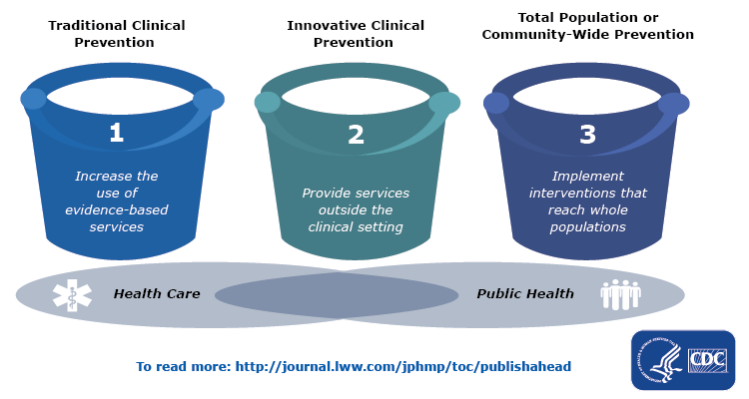
Centers for Disease Control and Prevention’s Three Buckets of Prevention.

Driven by payment policy changes, our health care system is transforming from one focused on episodic, nonintegrated care toward one that is value-based and would benefit from collaboration with allied community efforts. CDC developed a framework to conceptualize such integration across 3 areas of prevention— traditional clinical preventive interventions, interventions that extend care outside of the care setting, and population or community-wide interventions (11) (Figure 1). Although work in all of these areas is necessary to improve health, the work of Public Health 3.0 is focused on the second and third areas.

To improve the health of all people in America, we must also address factors *outside* of health care. Doing so means we must build on past successes and work across sectors to get closer to the essential definition of public health: *Public health is what we do as a society to ensure the conditions in which everyone can be healthy* (12).

## The Evolution of Public Health

This expanded mission of public health was underscored in the 1988 Institute of Medicine (IOM, now the National Academy of Medicine) report, *The Future of Public Health* (12). It is even more salient today. Pioneering communities across the country are demonstrating how this can be achieved, particularly when led by local public health departments (13).

The 2002 IOM report, *The Future of the Public’s Health in the 21st Century* (14), called for strengthening governmental public health capabilities and requiring accountability from and among all sectors of the public health system. However, public health has been significantly underfunded. Relative to health care spending, the United States has made paltry investments in upstream, nonmedical determinants of health, such as social services, education, transportation, environmental protection, and housing programs. This lack of investment has had detrimental effects on population health (15). In addition, the 2008 recession precipitated a large and sustained reduction in state and local spending on public health activities (16). In 2012, nearly two-thirds of the US population lived in jurisdictions in which their local health department reported budget-related cuts to at least one critical program area (17).

Unfortunately, the need to strengthen the public health system, and the peril for failing to do so, is often only revealed in the context of disasters and crises. For example, in the aftermath of Hurricane Katrina, it became apparent that restoring health care services alone was insufficient in restoring New Orleans’s health care system. The water crisis in Flint, Michigan, reminded us of the costly consequences of not placing health and environmental impacts at the center when making decisions that affect the public’s health. For a community to address fundamental drivers of health while establishing readiness and resilience to crises requires a strong public health infrastructure, effective leadership, useable data, and adequate funding.

## Public Health 3.0: A Renewed Approach to Public Health

**Figure 2:**
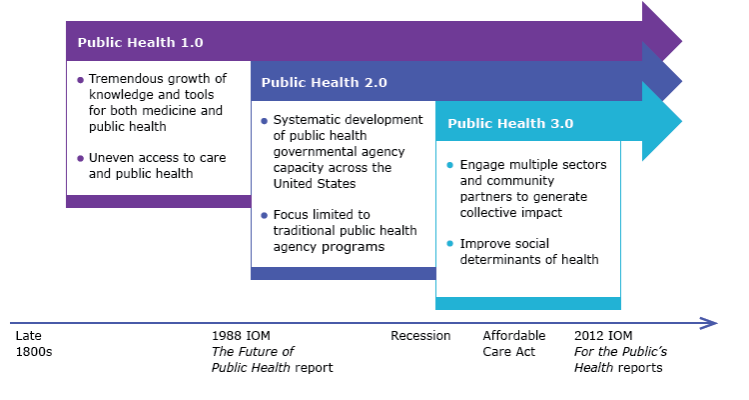
Evolution of public health practices. Abbreviation: IOM, Institute of Medicine.

Public Health 3.0 builds on the extraordinary successes of our past (Figure 2). *Public Health 1.0* refers to the period from the late 19th century through much of the 20th century when modern public health became an essential governmental function with specialized federal, state, local, and tribal public health agencies. During this period, public health systematized sanitation, improved food and water safety, expanded our understanding of diseases, developed powerful prevention and treatment tools such as vaccines and antibiotics, and expanded capability in epidemiology and laboratory science. This scientific and organizational progress meant that comprehensive public health protection — from effective primary prevention through science-based medical treatment and tertiary prevention — was possible for the general population.

*Public Health 2.0* emerged in the second half of the 20th century and was heavily shaped by the 1988 IOM report *The Future of Public Health* (12). In that seminal report, the IOM posited that public health authorities were encumbered by the demands of providing safety-net clinical care and were unprepared to address the rising burden of chronic diseases and new threats such as the HIV/AIDS epidemic. The report’s authors declared, “This nation has lost sight of its public health goals and has allowed the system of public health activities to fall into disarray.”

With this call to action, the IOM defined a common set of core functions, and public health practitioners developed and implemented target capacities and performance standards for governmental public health agencies at every level. During the 2.0 era, governmental public health agencies became increasingly professionalized.

*Public Health 3.0* refers to a new era of enhanced and broadened public health practice that goes beyond traditional public department functions and programs. Cross-sectoral collaboration is inherent to the Public Health 3.0 vision, and the Chief Health Strategist role requires high-achieving health organizations with the skills and capabilities to drive such collective action. Pioneering US communities are already testing this approach to public health, with support from several national efforts.

## Learning From the Field

At the core of Public Health 3.0 is the notion that local communities will lead the charge in taking public health to the next level and ensuring its continued success. Over the spring and summer of 2016, we visited communities across the United States to assess the accuracy of the 5 key components of the Public Health 3.0 framework and to hear firsthand what policy and other changes would support and sustain communities’ Public Health 3.0 work.

We selected 5 geographically and demographically diverse communities and convened listening sessions with approximately 100 participants each. Each meeting showcased successful multisectoral collaboration designed to address the social determinants of health. The communities visited were Allegheny County, Pennsylvania; Santa Rosa, California; Kansas City, Missouri; Nashville, Tennessee; and Spokane, Washington. They were selected as representative of the broader Public Health 3.0 movement because of their national reputation for multisectoral collaboration, evidence of a strong local public health leader, innovative use of data and metrics, and funding. They also had experience in public health department accreditation. Allegheny County, Pennsylvania, is a prototype for the model including their work to form a structured partnership supporting health and blending and braiding funding across several governmental jurisdictions (18).

In these listening sessions, local leaders shared their knowledge, strategies, and ideas for successfully implementing Public Health 3.0–style initiatives. Meeting participants represented an array of expertise beyond public health and health care. Although participants noted unique challenges and successes in each region, many common themes emerged across the meetings.

## Recommendations to Achieve Public Health 3.0

Based on insights gathered from the public health community at these listening sessions, from conversations with leaders, and from a review of prior reports that lay out a framework for strengthening public health, we propose 5 broad recommendations that define the conditions needed to support health departments and the broader public health system as it transforms into the Public Health 3.0 model. A more detailed list of specific actions can be found in the Appendix and in the full report (18).

1. Public health leaders should embrace the role of **Chief Health Strategist for their communities —** working with all relevant partners so that they can drive initiatives including those that explicitly address “upstream” social determinants of health. Specialized Public Health 3.0 training should be available for the public health workforce and public health students.
 
Although the local health officer often may serve in the role of Chief Health Strategist, there are circumstances in which such leadership comes from those in other sectors. Regardless, the public health workforce must acquire and strengthen its knowledge base, skills, and tools to meet the evolving challenges to population health, to be skilled at building strategic partnerships to bring about collective impact, to harness the power of new types of data, and to think and act in a systems perspective. This will require a strong pipeline into the public health workforce, as well as access to ongoing training and midcareer professional development resources.
 
2. Public health departments should engage with community stakeholders — from both the public and private sectors — to form vibrant, **structured, cross-sector partnerships** designed to develop and guide Public Health 3.0–style initiatives and to foster shared funding, services, governance, and collective action.
 
Communities should create innovative and sustained organizational structures that include agencies or organizations across multiple sectors and with a shared vision, which allows blending and braiding of funding sources, capturing savings for reinvestment over time, and a long-term roadmap for creating health, equity, and resilience in communities.
 
3. Public Health Accreditation Board (PHAB) criteria and processes for department **accreditation** should be enhanced and supported to best foster Public Health 3.0 principles, as we strive to ensure that every person in the United States is served by nationally accredited health departments.
 
As of August 2016, approximately 80% of the US population lived in the jurisdiction of one of the 324 local, state, and tribal health departments that has been accredited or is in the process of becoming accredited by the PHAB (19). The vision of ensuring that every community is protected by an accredited local or a state health department (or both) requires major investment and political will to enhance existing infrastructure. Although research found accreditation supports health departments in quality improvement and enhancing capacity (20), the health impact and return on investment of accreditation should be evaluated on an ongoing basis.
 
4. Timely, reliable, granular-level (ie, subcounty), and **actionable data** should be made accessible to communities throughout the country, and clear **metrics** to document success in public health practice should be developed to guide, focus, and assess the impact of prevention initiatives, including those targeting the social determinants of health and enhancing equity.
 
The public and private sectors should work together to enable more real-time and geographically granular data to be shared, linked, and synthesized to inform action while protecting data security and individual privacy. This includes developing a core set of metrics that encompass health care and public health, particularly the social determinants of health, environmental outcomes, and health disparities.
 
5. **Funding for public health should be enhanced and substantially modified**, and innovative funding models should be explored to expand financial support for Public Health 3.0–style leadership and prevention initiatives. Blending and braiding of funds from multiple sources should be encouraged and allowed, including the recapturing and reinvesting of generated revenue. Funding should be identified to support core infrastructure as well as community-level work to address the social determinants of health.
 
To secure sufficient and flexible funding in a constrained and increasingly tightening funding environment, local public health needs a concrete definition of the minimum capabilities, the costs of delivering these services, and a structured review of funding streams to prioritize mandatory services and infrastructure building.

## Early Action on the Recommendations

Upon the release of the report, several public and private organizations committed to advancing its recommendations. It was embraced by the American Public Health Association as the blueprint for the future of public health (21); others committed to developing training for Chief Health Strategists (22) or to building bridges between public health and the clinical care system, including payers (23). The US Department of Health and Human Services (HHS) implemented 3 priority recommendations, including extending reporting on accreditation status to federal public health entities, establishing a social determinants of health workgroup to support alignment of HHS policies, and launching a conversation about state-based opportunities to leverage health and human services resources to improve the public’s health (23). Additionally, CDC’s Health Impact in 5 Years (HI-5) initiative (24) provides nonclinical, community-wide toolkits to address social determinants of health that have demonstrated not only health improvement but also cost-effectiveness within 5 years. Community-level uptake and action through these resources could accelerate the impact of Public Health 3.0 collaborations.

## Key Barriers

For many communities, transforming to a Public Health 3.0 model will prove challenging. Although funding has stabilized, local health departments continue to face resource challenges from local financing streams, and proposals to reduce federal public health spending are likely to have a major impact at the local level (25). Despite promising advances such as the Big Cities Project, the absence of nonproprietary tools for data, analytics, metrics, and other uses leaves actionable information out of reach for most localities (25). Additionally, the daily challenges of meeting statutory public health responsibilities and a lack of experience and skill prevents most local health leaders from acting as Chief Health Strategists to bring people together across sectors. Finally, the basic foundational structure of local governmental public health may itself be a barrier to efficient and cost-effective coordination at the local level.

## Conclusion

The era of Public Health 3.0 is an exciting time of innovation and transformation. With the Public Health 3.0 framework, we envision a strong local public health infrastructure in all communities and its leaders serving as Chief Health Strategists that partner with stakeholders across a multitude of sectors on the ground to address the social determinants of health. With equity and social determinants of health as guiding principles, every person and every organization can take shared accountability to ensure the conditions in which everyone can be healthy regardless of race, ethnicity, gender identity, sexual orientation, geography, or income level. If successful, such transformation can form the foundation from which we build an equitable health-promoting system — in which stable, safe, and thriving community is a norm rather than an aberration. The Public Health 3.0 initiative seeks to inspire transformative success stories such as those already witnessed in many pioneering communities across the country. The challenge now is to institutionalize this expanded approach to community-based public health practice and replicate these triumphs across all communities, for the health of all people.

## Appendix Full List of Recommendations to Achieve Public Health 3.0.

**Leadership & Workforce**

- Public health associations such as Association of State and Territorial Health Officials (ASTHO) and National Association of County and City Health Officials (NACCHO) should develop best practice models and training for current public health leaders looking to work as Chief Health Strategists.
- The Health Resources and Services Administration (HRSA) should incorporate principles of Public Health 3.0 and social determinants of health in their workforce training programs, including the National Health Service Corps orientation, public health training center, and National Coordinating Center for Medicare and Medicaid Services Accountable Health Communities Model.
- Local public health agencies should partner with public health training centers and academic schools and programs of public health to inform training that meets the local public health workforce needs.
- The business and public health communities should jointly explore leadership development and workforce enrichment opportunities such as short-term fellowships or exchange programs, with a particular focus on the financial and operational capacity of local health departments.
- Academic institutions should encourage their faculty and administrations to develop meaningful partnerships with local public health departments and support service learning and internships for students from all disciplines in state and local health departments.
- Local health departments should train their leaders and staff in the concept and application of the collective impact model of social change.
- Public health should work with leadership institutes and business schools to establish professional development resources and opportunities.

**Strategic Partnerships**

- Local public health agencies should form cross-sector organizational structures aimed at achieving a collective vision of community health that are capable of receiving and sharing resources and governance.
- The US Department of Health and Human Services (HHS) should work with others to develop a report defining the key characteristics of successful local public health models that address social determinants of health through cross-sector partnerships and recommending pathways to wide adoption.
- The Assistant Secretary for Preparedness and Response (ASPR) and the Centers for Disease Control and Prevention (CDC) should work with state and local health entities to ensure synchronization between health care practices, coalitions, and public health entities. Pre-crisis collaboration is essential to improve sharing of limited resources, improve timely and accurate communication, and improve sharing of data relevant to preparedness planning and response.
- Local public health leaders should create cross-jurisdictional organizational structures or partnerships for community development efforts.
- Public health entities should partner with environmental health agencies to address the environmental determinants of health.
- HHS should continue to develop tools and resources (such as the HI-5 [Health Impact in 5 Years]) that identify system-level drivers of health disparities, connecting health and human services, and work with communities to translate evidence to action.
- HRSA should recommend that health centers document collaboration with their state and/or local health department.
- Health care providers should identify clear mechanisms to engage with local public health as part of their effort to achieve the three-part aim of better care, smarter spending, and healthier people.
- The Centers for Medicare and Medicaid Services (CMS) and ASPR should work together to ensure state and local public health entities engage health care providers during times of crisis or disaster. Preparedness measures are essential to healthier and more resilient people.
- The Substance Abuse and Mental Health Services Administration should encourage state mental health and substance use disorder agencies and other grantees to collaborate with state, local, and tribal public health entities in achieving PH3.0 goals.
- The Agency for Healthcare Research & Quality should ensure linkages between primary care and public health via the Primary Care Extension Program and evaluate outcomes.
- The National Institutes of Health should continue its community participatory research and engagement efforts, such as the Clinical and Translational Science Awards and the Partnerships for Environmental Public Health, to accelerate translation of evidence to community action, as well as to generate new knowledge in the evaluation and implementation of public health interventions.
- Public health leaders should pursue local partnerships to ensure population health is central in all community development efforts.

**Infrastructure and Accreditation**

- HHS should assess opportunities to incentivize Public Health Accreditation Board (PHAB) accreditation through federal programs and policies.
- HHS should require state and local health departments receiving federal grants to indicate their PHAB accreditation status, including applications in progress or plans to apply in the future.
- The federal government should partner with the private sector to create a learning community for local health departments seeking to engage in PH3.0 work with a particular focus on collective impact models to address the social determinants of health.
- Resources to support the accreditation process and maintenance should be more readily available from public and private funding sources.
- PHAB should continue to evolve accreditation expectations by incorporating Public Health 3.0 concepts.
- Philanthropic organizations supporting local public health activities and social interventions should require grant applicants to collaborate with local health departments.
- ASTHO and NACCHO should accelerate their support of state and local health departments moving to accreditation.
- PHAB and its strategic partners should continue to enable pathways to accreditation for small and rural health departments.
- States should assess the efficiency and effectiveness of their local health departments, including addressing jurisdictional overlaps and exploring opportunities for shared services mechanisms.

**Data, Metrics, and Analytics**

- HHS should utilize opportunities such as Healthy People 2030, NCVHS’s population health subcommittee, the Evidence-Based Policymaking Commission, and the census to elevate metrics related to social determinants to be leading health indicators, to define community-level indicators that address the social determinants of health and to explore models to leverage administrative data.
- NCVHS should advise the secretary of HHS to incentivize the integration of public health and clinical information.
- CDC should continue its work with the private sector to make subcounty-level data including health, health care, human services, environmental exposure, and social determinants of health available, accessible, and useable.
- HHS should work with public health leadership and the private sector to develop a nonproprietary tool to support geographic information systems and other analytic methods for front-line public health providers.
- Health systems and other electronic health data repositories should prioritize data sharing at the federal, state, and local level with the goal of achieving a learning health system inclusive of public health by 2024 as described in the Office of the National Coordinator for Health Information Technology (ONC) Nationwide Interoperability Roadmap.
- The HHS Office for Civil Rights should continue to develop guidance for the public health system to provide clarity on private and secure data use, as well as guidance to promote civil rights compliance to address those social determinants which are the product of discriminatory practices.
- ONC and the Administration for Children and Families should continue to establish clear data and interoperability standards for data linkage between health and human services sectors.
- HHS should continue to identify gaps in the collection of data relating to race/ethnicity, language, gender identity or sexual orientation in existing surveys. When feasible, governmental and nongovernmental stakeholders at all levels — federal, state, local, and tribal — should collect standardized, reliable data concerning disparities.
- HHS should facilitate linking environmental and human services data to health.

**Sustainable and Flexible Funding**

- The CMS and private payers should continue to explore efforts to support population-level health improvements that address the social determinants of health.
- HHS should explore transformation grants for state and local health departments to evolve toward PH3.0 structure, analogous to the State Innovation Model (SIM) grants to support health care system transformation.
- State governments receiving funds through SIM or Medicaid Waiver processes should be required to document their health department accreditation status and their strategies for addressing the social determinants in partnership with their local public health departments.
- States should maximize their use of the funding through the Health Services Initiative option under the Children’s Health Insurance Program to advance their public health priorities for low-income children.
- HHS should enhance its coordination both within the department and with other agencies, developing and executing cross-agency efforts to strategically align policies and programs that address the social determinants of health.
- Public and private funders should explore options to provide more flexibility for accredited health departments to allocate funds toward cross-sector efforts including partnership development and collective impact models in addressing the social determinants.
- Communities should examine how to best use the Affordable Care Act’s community benefits requirement for nonprofit hospitals by coordinating the alignment of the data collection process and pooling resources and how these can be used to advance and provide funding for public health.
- Public health agencies and academic institutions should periodically calculate the funding gap — the difference between the costs of providing foundational capabilities by each local health department and its current funding level — and communicate these figures in the context of forging partnerships and expanding funding sources.
